# Microchannel Structural Design For a Room-Temperature Liquid Metal Based Super-stretchable Sensor

**DOI:** 10.1038/s41598-019-42457-7

**Published:** 2019-04-11

**Authors:** Qinwu Gao, Hui Li, Jinjie Zhang, Zhenwen Xie, Jinyong Zhang, Lei Wang

**Affiliations:** 1 Shenzhen Institutes of Advanced Technology, Chinese Academy of Science, Shenzhen, 518055 China; 2 Jiangsu Provincial Key Laboratory of Advanced Photonic and Electronic Materials, Collaborative Innovation Center of Advanced Microstructures, School of Electronic Science and Engineering, Nanjing University, Nanjing, 210093 China

## Abstract

Room-temperature liquid metal has been widely used in flexible and stretchable sensors, focusing on embedding liquid metal in microchannels, liquid metal microdroplets formation, captive sensors, and liquid metal nanoparticles, etc. In this paper, a facile Eutectic Galium-Indium (EGaln) liquid-based microfluidic high-sensitivity, skin-mountable, and ultra-soft stretchable sensor is developed. It comprises Ecoflex microfluidic assembly filled with EGaln, which serves as the working fluid of the stretchable sensor. The lithography method is applied to achieve microfluidic channel. The microfluidic channel is optimized by using topology method and finite element analysis, making this device with high conformability and high stretchability. This method achieved an outstanding effect on elastomer-encapsulated strain gauge, which displays an approximately linear behavior with a gauge factor (GF). The GF could reach as high as 4.95 when the strain ultimately reached 550%. Applications of detection of the joints, fingers, and wrists has been conducted and showed excellent results. This work can further facilitate the exploration and potential realization of a functional liquid-state device technology with superior mechanical flexibility and conformability.

## Introduction

Flexible and stretchable sensors have attracted substantial attention due to their unique characteristics, such as low modulus, light weight, high flexibility, and stretchability^1–6^. And flexible and stretchable sensors are widely used in emerging applications. These include soft robotics^7^, wearable consumer electronics^8,9^, e-skins^10,11^. As the demands for these applications increase, the requirements for flexible and stretchable sensors will be more stringent^12^. However, traditional metallic and semiconducting sensors only withstand very limited stretchability before fracture. Thus, they are not suitable for stretchable applications^13,14^. Aimed at this problem, diverse methods and strategies are proposed to achieve the stretchability. Some strain sensors used structuring of easy to stretchable, such as spring, buckling, wave^15–19^. The stretchability of the vast majority of sensors is achieved by embedding, depositing, and printing the conductive material and the substrate^20–24^. Diverse flexible and stretchable substrates^25,26^, novel mechanically durable materials^27–29^, deformable electrodes and novel processing technology^30,31^ is promising for the development of stretchable sensors in the near future driving innovations in stretchable sensors for emerging applications at a tremendous pace.

However, these sensors are not able to remain functional at strains over 100%, which limited by large-scale stretchability occasions. At the same time, material delamination and/or local fracture in rigid electronic components are frequently observed in stretchable sensors. The main reason for the poor durability is that there is an intrinsic difference in the Young’s modulus between the rigid conductors and the soft support material. To solve the problem of Young’s modulus mismatch, many materials have been explored. Liquid conductors have attracted substantial attention^32–35^, due to their advantages of low Young’s modulus and high durability, which are maintained even under a large strain. Awareness has grown among researchers that using softer liquid materials to generate stretchable conductors is a promising method to fabricate high-performance stretchable sensors. Wenlong Cheng fabricated reliable and long-term stretchable sensors using ionic liquids, which overcome the mechanical-mismatch problem^31^. A particular type of conductive liquid material that is eutectic gallium-indium (EGaln) come into people’s perspective^35–41^. EGaln is an alloy of gallium and indium that can maintain a liquid state at room temperature. Due to high surface tension and high electrical conductance^42^, EGaln is an ideal conductor for a stretchable and flexible sensor. However, the stretchable and flexible sensor with EGaln conductor also exist a drawback that is low gauge factor(GF)^31^. Here, we explore the GF influence factors, and design a reasonable microfluidic channel to increase the GF of stretchable and flexible sensor. And then the lithography technology is applied to establish a relative smooth microfluidic channel. The way of EGaln is injected into the microfluidic channel of Ecoflex elastomer is used to offer a sensor with high stretchability, high conformability.

## Production Procedure

**Figure 1:**
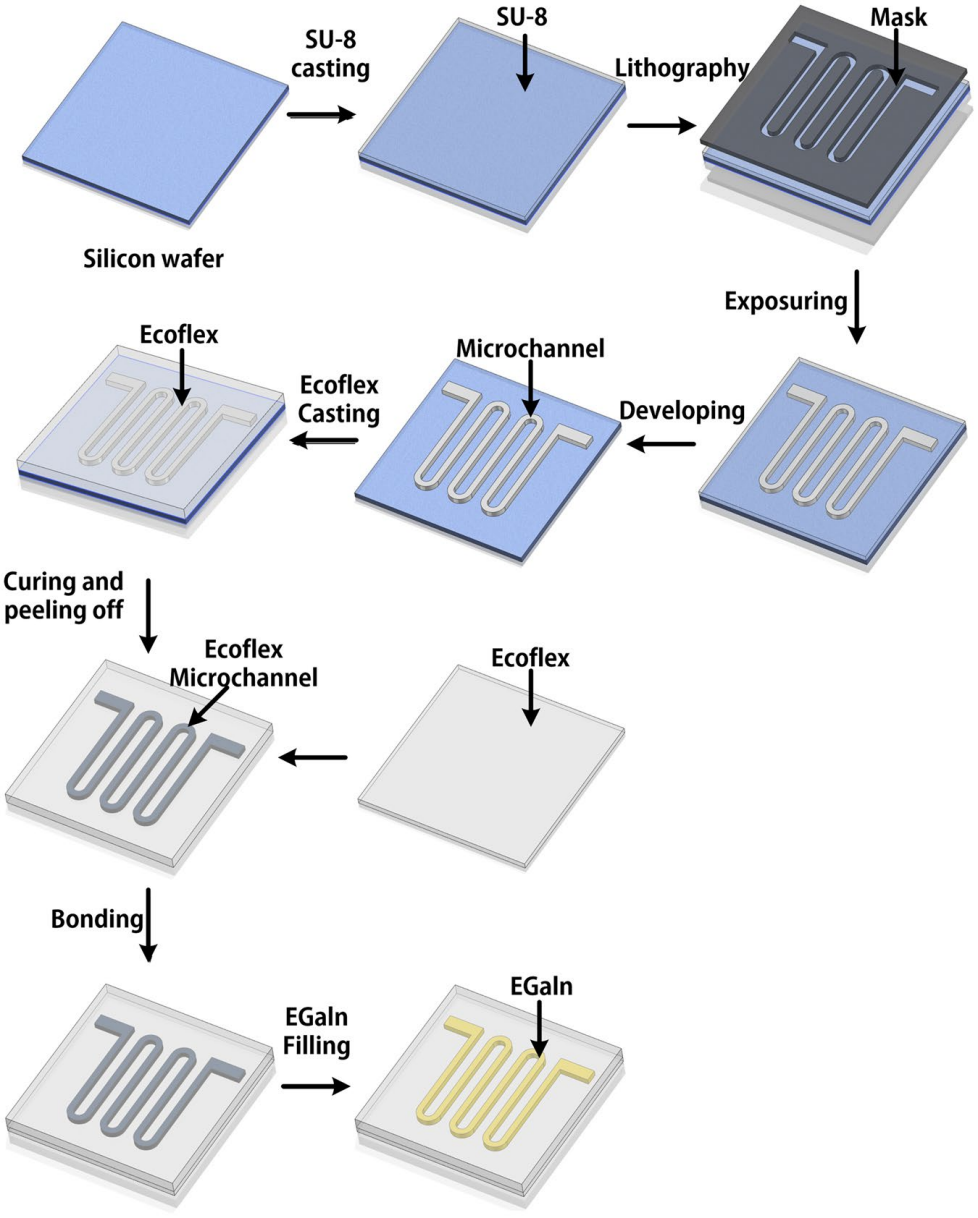
Schematic illustration of the fabrication of the highly flexible and stretchable EGaln microchannel.

The entire production process is shown in Fig. 1 below. At first, the SU-8 2100 negative photoresist was spin-coated on the silicon wafer by 500 rpm (ramp rate of 100 rmp/s) for 7 s, followed by 1500 rmp (ramp rate of 100 rmp/s) for 60 s. This was followed by pre-exposure baking at 65 for 5 min and 95 for 30 min. Then the SU-8 layer was patterned using ultraviolet (UV) lithography by mask aligner (Karl Suss MJB3) for 53 s (130 mj/cm^2^ exposure energy). Samples were post-exposure baked at 65 for 5 min and 95 for 15 min. After cooling to room temperature, samples were soaked in SU-8 developer (MicroChem®) to chemically remove uncrosslinked material from the SU-8 layer. Next, Ecoflex was spin-coated on the silicon wafer with microfluidic channel and heated by heating plate at 60 °C. After the Ecoflex has solidified, the Ecoflex was peeled from the silicon wafer. Used same method fabricated Ecoflex flexible substrate without microfluidic channel. And then two Ecoflex flexible substrate was adhered to together. In last, the EGaln was injected in the microfluidic channel of the Ecoflex flexible substrate.

**Figure 2:**
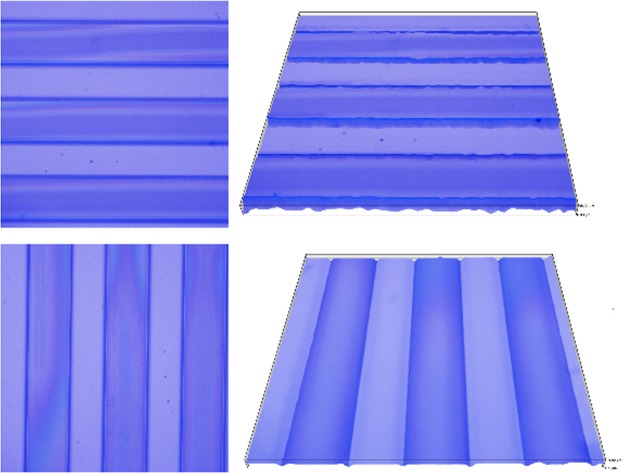
Photo of SU-8 microchannel on the silicon wafers fabricated by lithography.

A microfluidic channel was created; optical micrographs of the patterns are shown in Fig. 2. Using this method, we were able to create microfluidic channel with high resolution crescent, which can meet the requirement of the size and flatness.

## Results and Discussion

### Device design and working mechanism

This flexible and stretchable sensor operates based on a deformability-dependent resistive sensing mechanism, which can be described by Equation 1^42^

1 $${\rm{\Delta }}R=R-{R}_{0}=\rho \frac{L+{\rm{\Delta }}L}{(\omega +{\rm{\Delta }}\omega )(h+{\rm{\Delta }}h)}-\rho \frac{L}{\omega h}$$

**Figure 3:**
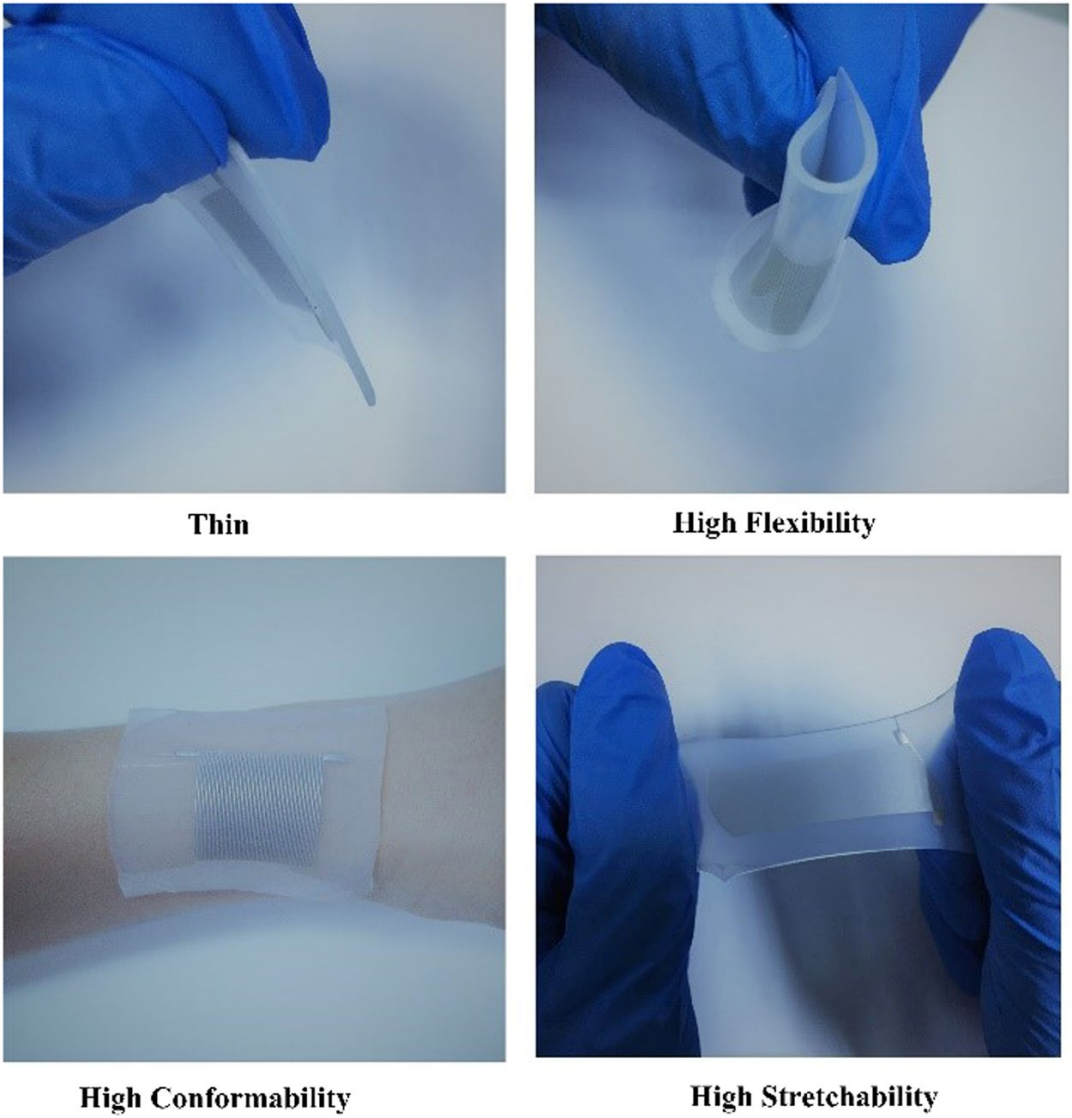
Image of liquid-based microfluidic stretchable sensor with outstanding property for flexibility, conformability and stretchability.

Where *R* and *R*_0_ are the resistances of the microchannel when stretched by Δ*L* and not stretched, respectively, $\rho$ is the electrical resistivity of EGaln, *L* is the length of the microchannel, and $\omega$ and *h* are the width and height of the cross-section of the microchannel, respectively. When the surface of the device is stretched, the microfluidic channel experiences a minute stretchability deformation, this leads to a decrease the cross-sectional area of the channel and increase the length of the channel which, in turn, results in an increase in the resistance of the EGaln across the microfluidic channel. However, upon the release of the external pressure and due to the elastic property of the Ecoflex, the microfluidic channel will recover to its original state. At the same time, the resistance value will return to the initial state. In this work, the specific increase and decrease in the electrical resistance of the EGaln correspond to the characteristic response of different mechanical forces applied on the tactile sensor. The actual fabricated tactile sensing device is shownin Fig. 3. Generally, EGaln liquid based flexible microfluidic flexible and stretchable sensor exhibits several distinctive features, such as superior thinness, high flexibility, large area conformability, and small physical size.

### Mechanical deformation characterization

**Figure 4:**
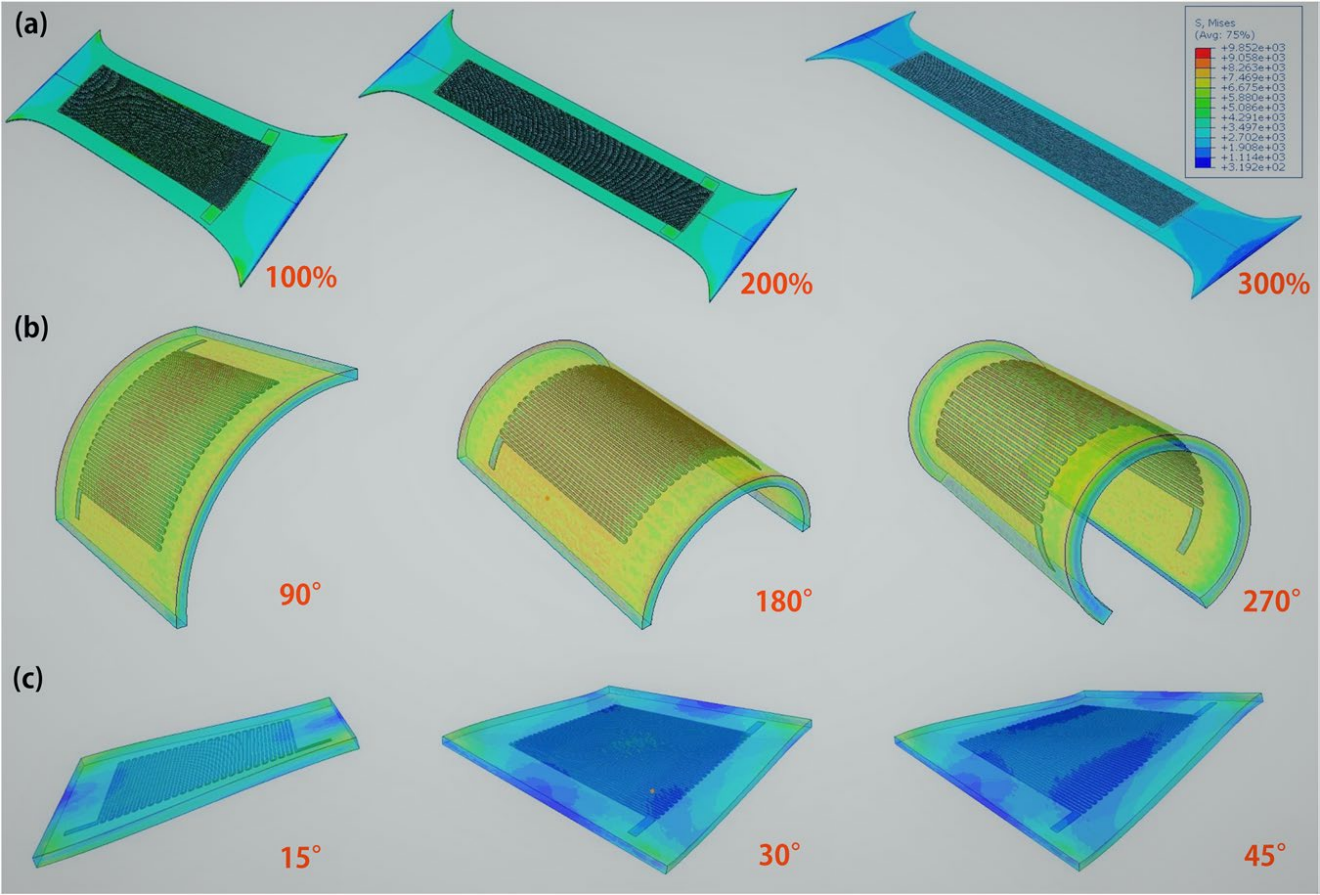
Schematic diagram of liquid-based microfluidic stretchable sensor on condition of stretchability, bend and wrinkle. (**a**) Schematic diagram of liquid-based microfluidic stretchable sensor tensile force corresponding to 100%, 200% and 300%. (**b**) Schematic diagram of liquid-based microfluidic stretchable sensor bending force corresponding to angle of 90°, 180° and 270°. (**c**) schematic diagram of liquid-based microfluidic stretchable sensor fold force corresponding to angle of 15°, 30° and 45°.

To execute its designated functions robustly and effectively, EGaln microchannel sensor need to have feature of high degree of flexibility, conformability and stretchability as well as be capable of withstanding a wide range of mechanical deformation, such as stretching, bending and twisting. In order to understand the performance of EGaln microchannel sensor, the force distribution of EGaln microchannel sensor corresponding to stretching, bending and twisting is analyzed by the analogue simulation. Figure 4 show the force distribution condition. The force distribution uniformly of device when stretched. Even if the stretchability up to 300%, this sensor don’t appear the high stress condition, which can prevent fatigue failure, making the sensor with a high stretchability. This explain the microchannel designed reasonable that don’t damage structure strength of the flexible substrate. The microchannel show uniformity deformation when stretched, contributing to create outstanding linearity sensor and establish the relationship of deformation and resistance change. What’s more, this device don’t appear high stress when bent and twisted, which well-illustrates this device can achieve the designated function robustly and effectively, not making the function failures due to high stress fatigue damage.

**Figure 5:**
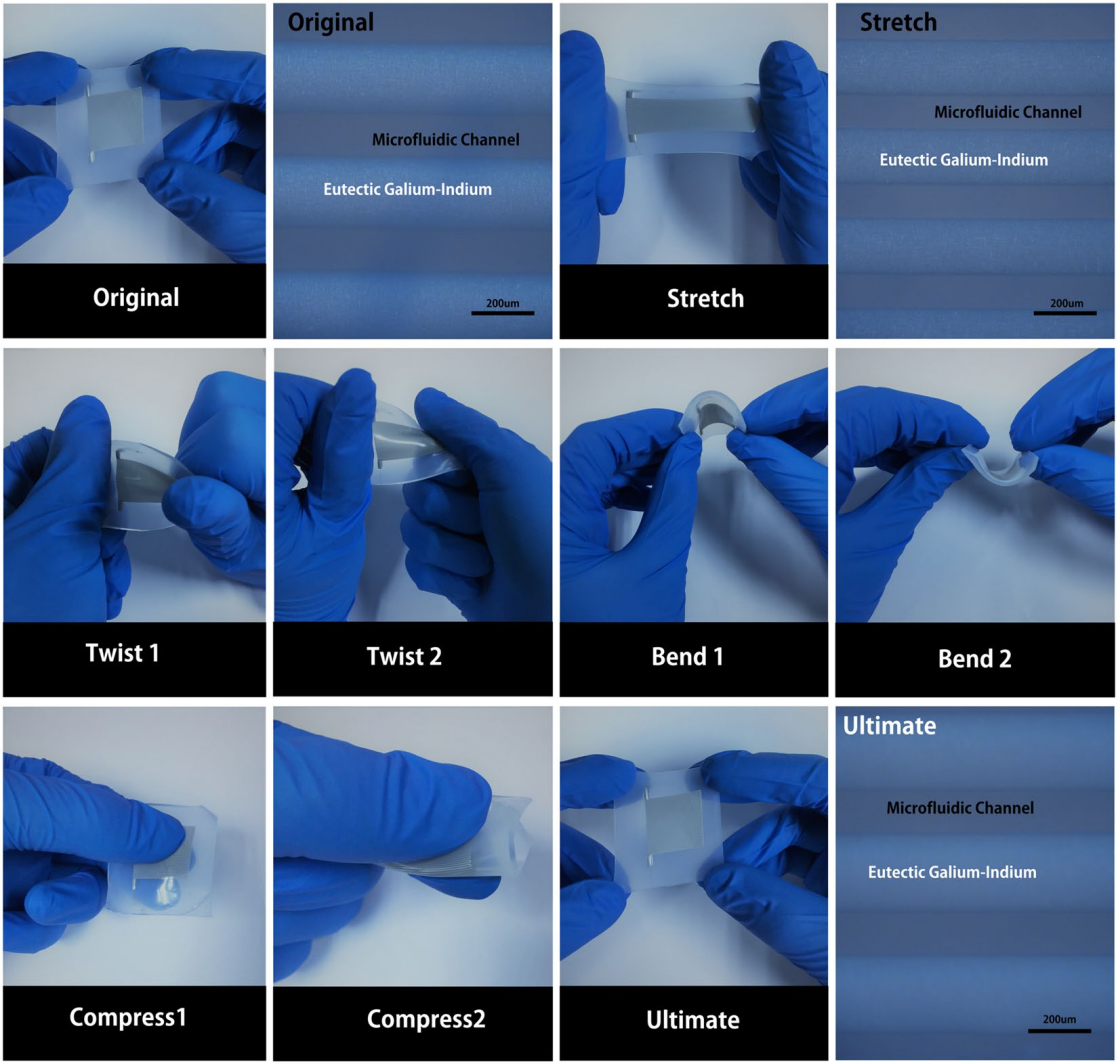
(**a**) Photograph of the device with no applied deformation (left) with the corresponding microscopy image of the EGaln in microfluidic channel (right). (**b**) Photograph of the device being stretched in a direction parallel to the channel (left) with the corresponding microscopy image showing that the EGaln confinement was well maintained within the channel (right). (**c**) Photographs of the device as it underwent different forms of user applied deformation: twisting, bending and compressing. (**d**) Photograph of the same device after it went through all the different mechanical deformations (left) with the corresponding microscopy image showing the integrity of the EGaln confinement within the microchannel (right).

To further evaluate and quantify the structural strength of flexible EGaln microchannel sensor, the device is handled by loading test and meticulous observe the change of the microchannel. Figure 5 show the flexible EGaln microchannel sensor without experiences loading test and EGaln being confined within the channels without leakage. Then the flexible EGaln sensor was stretched several times by the parallel direction, in addition to the cross section area get small without any failure modes. Then flexible EGaln microchannel sensor was loaded by the way of bending and twisting in different direction and angle several times. The experimental result depicts that the channel with well-maintained structure without cross section area change, edge coarse and leakage. This express flexible EGaln microchannel sensor with outstanding mechanical stability even if on condition of experience several forms of mechanical deformations. In other words, as a liquid state device, flexible EGaln microchannel sensor displayed excellent mechanical deformability with superior working fluid confinement. It is worth noting that several bonding forms for Ecoflex was explored to enhance working fluid confinement. Three distinct bonding strategies respectively are thermal bonding, ozone treatment and oxygen plasma treatment that seem to not achieve the ideal effect for Ecoflex bonding. On condition of performing many groups experiments, two Ecoflex have not bonded together successfully. Aimed this condition, the room temperature solidification was used to bond two Ecoflex. Firstly, the Ecoflex was spin-coated on the silicon wafer with microchannel, the Ecoflex was peeled from the silicon wafer after the Ecoflex is in the complete solidification. Another Ecoflex is was spin-coated on the silicon wafer without microchannel for 25 min, which the Ecoflex is in the condition of half solidification that put on bonding not only damage the channel structures, but also complete bond together. Due to the two Ecoflex substrates complete bond together, the peel test by pulling the two substrates don’t separate the two Ecoflex substrates.

### Performance analysis

This sensor has already demonstrated preeminent mechanical performances which suffered no structural damage on condition of high strength mechanical charging. The flexible EGaln microchannel sensor is device that to accomplish design functions. It isn’t not enough that this sensor only has high mechanical durability. The sensor working mechanism is the change of resistance corresponding to the change of the cross-sectional area of the microchannel as the sensor stretched. The cross-sectional area of the microchannel of this sensor gets smaller when stretched, leading to the resistance gets bigger. In general, it is helpful to accomplish design functions that more obvious increased resistance value and outstanding linear relationship of increased resistance value and strain. The response characteristic of flexible EGaln microchannel sensor was conducted. The Gauge factor is one important index to measure the sensitivity of strain sensors, which can be determined by Equation 2.

2 $$\mathrm{GF}=\frac{{{\rm{\Delta }}{\rm{R}}/{\rm{R}}}_{{\rm{O}}}}{{\rm{\varepsilon }}}$$

**Figure 6:**
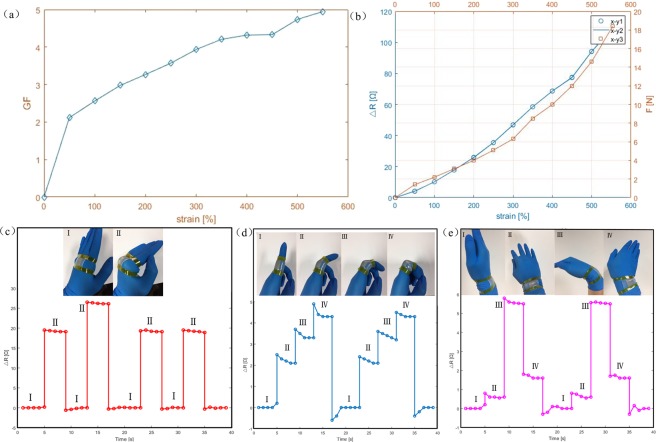
(**a**) The relationship between GF and strain. (**b**) The relationship of resistance change and strain (blue lines), x-y1 and x-y2 signify first stretchability and fiftieth stretchability. Photograph of relationship of tensile force and strain (orange line), x-y3 signify the condition of the tensile force and strain. Photographs of this sensors attached on the joint (**c**)/fingers (**d**)/wrists (**e**) of different motions, and corresponding resistance change.

Where GF is the gauge factor of the stretchable sensor. ΔR is the change of the sensor’s resistance when stretching. R_o_ is the sensor’s original resistance without any stretch. ε = ΔL/L_o_, which is the sensor strain. ΔR is the change of the sensor’s length when stretching. R_o_ is the sensor’s original effective length without any stretch. According to the calculation, the GF could reach as high as 4.95 when the sensor was ultimately stretched to 550%, and it was stretched broken nearly reaching 590%, which showed excellent sensitivity for the EGaln based sensors. The detailed relationship between GF and strain is shown in Fig. 6(a). Besides the outstanding sensitivity, some properties including comfortableness, repeatability, etc., are also essential effects for the wearable sensor to satisfy the requirements of human monitoring. According to the repeatable experiments, the sensor just generated minor stress change even through suffer large-scale stretch. Line x-y3 in Fig. 6(b) signify the condition of the tensile force and strain, which can decrease discomfortableness caused by excessive stress. Line x-y1 and Line x-y2 in the Fig. 6(b) signify first and fifth stretchability curve respectively. These two lines are almost overlapping. which shows the excellent repeatability of the sensor in case of multiple stretches. The great repeatability not only can offer accuracy monitoring parameter for subsequent analysis after several times uses, but also can satisfy human monitoring during movements.

To further demonstrate the reliability and practicality of flexible EGaln microchannel sensor, we attached this device to our human hand and probed its capability in recognizing different hand gestures. As a proof of concept of its application in detecting and differentiating various hand muscle induced motions, the tactile sensor was attached to the finger, wrist and joint and its dynamic pressure responses were monitored while different hand motions were executed. Figure 6(c) shows that the great change of sensor resistance will be chance when stretched. This result proved this sensor with outstanding strain performance, making it is easy to recognize the gesture of hand. There is no question for detecting the large-scale tensile strain. At the same time, the tensile scale will be gradually decreased, the change of sensor resistance still effective as shown in Fig. 6(d). The resistance make corresponding changes with the different bending angle and the resistance presents periodic to some extent, which explains this sensor with outstanding fatigue resistance and can pick up loading for a long time before fracture. What’s more, this sensor was applied on the wrist that can realize different rotary angles, the different rotary angles corresponding to different resistance change value, from the other way to explain this sensor with highly sensitivity that can catch angular variation accurately as shown in Fig. 6(d).

## Conclusion

We have demonstrated a flexible and stretchable EGaln microchannel sensor. The use of EGaln as the sensing element provides high sensitivity corresponding at the large-scale stretchability and EGaln as flexible substrate provides strength supporting of large-scale stretchability. The method of lithography was applied to establish channel that meet the requirement of size and flatness. Using the advance bonding way to stick two Ecoflex together, contributing to EGaln microchannel sensor with excellent mechanical deformability and superior working fluid confinement. Furthermore, this method is applied to the fabrication of an elastomer-encapsulated strain gauge that displays an approximately linear behavior. The gauge factor can reach as high as 4.95 when the strain reached the ultimate 550% strain,, which enormous increase value of gauge factor for the flexible sensor based on EGaln as conductor. This kind of sensor is easy to follow human skin mechanical deformations of bending and twisting that show the outstanding potential for emerging applications.
